# Prevalence of Risk Factors of Chronic Rhinosinusitis With Nasal Polyps Among the Saudi Population

**DOI:** 10.7759/cureus.45420

**Published:** 2023-09-17

**Authors:** Ahmad K Alnemare, Abdulaziz B Almutairi, Amirah F Almutairi, Turki Bin Mahfoz, Shaden B Almutairi, Athari K Alnemare, Rakan B Almjlad, Maathir N Alhumam, Raghad E Alghassab

**Affiliations:** 1 Department of Otolaryngology, College of Medicine, Majmaah University, Al-Majmaah, SAU; 2 College of Medicine, Majmaah University, Al-Majmaah, SAU; 3 Department of Otolaryngology, Faculty of Medicine, Al Imam Mohammad Ibn Saud Islamic University (IMSIU), Riyadh, SAU; 4 College of Medicine, King Faisal University, Al-Ahsa, SAU; 5 College of Medicine, Al Jouf University, Al-Jouf, SAU

**Keywords:** ent - ear nose and throat, prevalence, rhinosinusitis, risk factors, nasal polyps

## Abstract

Background

The burden of chronic rhinosinusitis symptoms experienced by Saudi citizens is considered an important factor in getting an overall insight of the problem in the region, therefore our study aimed to determine the prevalence of risk factors of chronic rhinosinusitis with nasal polyps and correlate the risk factors with various sociodemographic parameters among Saudi population.

Methods

This study analyzed five-year cross-sectional data from 2017-2021. A total of 386 participants were enrolled and subsequently divided into four groups: aged 18-25 years, 26-35 years, 36-45 years, and > 45 years. A study was conducted utilizing non-probability sampling targeting a population of Saudi nationality living in selected regions of Saudi Arabia. Data was collected through an online questionnaire which had been distributed through social media and had been analyzed accordingly using a statistical package for social sciences.

Results

In this study, 272 (70.5%) participants were female and 114 (29.5%) were male. A total of 374 (96.9%) participants were Saudis while 12 (3.1%) were non-Saudis. Risk factors were compared by age and gender of study participants. Having polyps in the nose (p-value 0.016) and a family history of polyps (p-value 0.049) showed a significant association with the gender of study participants. The frequency of having nasal polyps was significantly higher among male participants, however, having a family history of nasal polyps was significantly higher among female participants. The educational status of study participants showed a significant association with the use of cortisone (p-value 0.032) and having a broken nose (p-value 0.032). Having a family history of nasal polyps showed a significant (p-value 0.017) association with the socioeconomic status of study participants.

Conclusion

Nasal polyps are more common in males than females, however, having a family history of nasal polyps was significantly higher among female participants.

## Introduction

Inflammation of the nasal cavity and paranasal sinuses that is medically refractory after at least three weeks of maximal medical therapy, with at least two identifying symptoms (facial pain, nasal discharge, nasal obstruction, reduced smell), and endoscopic or radiographic disease confirmation, is referred to as chronic rhinosinusitis (CRS). CRS is further divided into CRS with nasal polyps (CRSwNP) and CRS without nasal polyps (CRSsNP), both of which are thought to have distinct histologic and pathophysiologic characteristics as well as hypothesized separate origins [1].

Two to four percent of individuals have CRSwNP, a chronic inflammatory disease of the nose and paranasal sinuses [2-4]. A typical clinical picture of patients with CRSwNP and comorbid asthma is characterized by older age, higher incidence of allergic rhinitis, longer duration of nasal symptoms, higher computed tomography (CT) and endoscopy scores, higher number of sinonasal surgeries, and bronchial obstruction [5,6]. Nasal polyps often manifest as bilateral inflammatory lesions that extend into the nasal airway from the ethmoid sinuses and below the middle turbinate. The presence of isolated nasal lesions medial to the middle turbinate, on the other hand, raises suspicion of cancer. Patients under 20 or over 80 years old who have suspected nasal polyps enhance the possibility of other clinical problems. Cystic fibrosis in children becomes a problem and unilateral nasal growths raise the possibility of an encephalocele [7,8]. Adults may have neoplasms if new polyps appear at an advanced age or in an atypical location. Despite the great frequency of CRS, it is still unknown what factors contribute to its etiology and its link to asthma. The aims of this study were to investigate the prevalence of risk factors of chronic rhinosinusitis with nasal polyps among citizens, to correlate the risk factors with various sociodemographic parameters, and to evaluate the burden of symptoms that are experienced by study participants.

## Materials and methods

This study analyzed five-year cross-sectional data from 2017-2021. A total of 386 participants were enrolled and subsequently divided into four groups: aged 18-25 years, 26-35 years, 36-45 years, and > 45 years.

Utilizing non-probability sampling targeting a population of Saudi nationality living in selected regions of Saudi Arabia, data were gathered using online questionnaires.

Prior to responding, participants had to complete an informed consent form. The Research Ethics Committee approved the study. By not demanding the separation of information from study respondents, not using this information for anything other than research, and not disclosing any information to other parties, the confidentiality and privacy of the information acquired in the research study were guaranteed.

Inclusion criteria included CRSwNP that was confirmed by a rhinologist according to the criteria of the Rhinosinusitis and Nasal Polyps guidelines. All patients with stable asthma and recorded asthma control test (ACT) scores ≥ 20 were included as well.

As for the exclusion criteria, patients who fulfilled any of the following requirements were disqualified from the study: patients who had systemic steroid treatment throughout the next four weeks; got biologic or immunosuppressive medication; had been given a diagnosis of chronic obstructive pulmonary disease (COPD) or another lung condition such as interstitial lung disease, pneumonia, lung cancer, allergic bronchopulmonary aspergillosis, or chronic obstructive pulmonary disease; had an autoimmune condition or immunodeficiency; had nasal disorders that would have impacted the study's findings, such as inverted papilloma or fungal sinusitis; and had considerable renal or liver impairment in addition to serious cardiac failure.

The questionnaire was divided into two main sections, the first of which collected data on demographics and the second of which focused on chronic rhinosinusitis with nasal polyps. Excel sheets were used to store the data, which was then analyzed using SPSS version 25 (IBM Corp., Armonk, NY, USA). Statistical analysis with a p-value of 0.05 was used to do both descriptive analysis and measures of association.

## Results

In this study, 272 (70.5%) of participants were females and 114 (29.5%) were males. Almost all participants (374, 96.9%) were Saudi while 12 (3.1%) were non-Saudi. The most frequent age group in this study was 18-25 years (45.3%) followed by 26-35 years old participants (21.2%), 36-45 years (16.6%), and lastly, >45 years old (16.6%) participants in which their frequency was low. Around 66.6% of the participants had bachelor’s degrees, 23.1% passed high school and only 6.2% had post-graduate education. The social status of participants showed that 30.6% were employed, 7.8% were retired, 37.3% were students and 24.4% were unemployed. Most of the participants were from the central Kingdom of Saudi Arabia (KSA) region (32.6%), 33.7% were from the eastern region, 2.6% were from the northern region, 6.2% were from the southern region and 24.9% were from the western side of KSA.

Table 1 describes the burden of symptoms among study participants. About 96% of the participants reported that they can distinguish between smells. However, 4% reported that they cannot distinguish between smells and 44% reported that this problem is of more than a year duration and 56% reported having this problem intermittently. Only 19% of the participants reported that medicine improves their perception of a smell. Among participants, 37% reported that their nose gets stuffy, 20% reported that they get cold frequently, 8% reported that they had asthma, 15% reported the use of cortisone, and 6% reported that they noticed symptoms after taking aspirin. Most of the participants (84%) used cortisone via nasal spray. Among the participants, 44.8% reported that they use medicine for their noses, and 78% reported that they use nasal decongestants. Eight percent of the participants said that they had nose surgery. Six percent of the participants reported that they had a broken nose, 8% reported that they had nasal polyps and 41% reported that they had a family history of polyps (Table 1).

**Table 1:** Burden of symptoms among study participants (n=386)

		N	%
Distinguish smells?	No	16	4
	Yes	370	96
Since When			
1-6 Months		8	50
<1 Month		1	6
>1 Year		7	44
Problem			
Intermittent		9	56
Ongoing		7	44
Is there a medicine that improves your perception of smells?			
Yes		3	19
No		13	81
Nose gets stuffy frequently	No	244	63
	Yes	142	37
Since When			
1-6 Months		26	6.7
7-12 Months		10	2.6
Less than one month		27	7.0
More than one year		79	20.5
Get cold frequently?	No	309	80
	Yes	77	20
When does it occur?			
Only in winter		26	34
Whatever season		51	66
Do you have asthma?	No	355	92
	Yes	31	8
If the answer is (yes), does taking aspirin cause or exacerbate it?			
Yes		8	26
No		23	74
Notice any symptoms after taking aspirin?	No	363	94
	Yes	23	6
Have you ever used cortisone for your nose?	No	328	85
	Yes	58	15
(yes), by which method did you use it?			
Oral Pills		9	16
Nasal Spray		49	84
Do you sometimes use any medicines for your nose?	No	213	55.2
	Yes	173	44.8
If yes then which kind of medicine			
Nasal decongestants		135	78
Others		38	22
Have you ever had nose surgery?	No	357	92
	Yes	29	8
If the answer is (yes), please specify the reason:			
Nasal polyps		17	59
Rhinoplasty		8	28
Sinusitis		4	14
Have you ever had a broken nose?	No	363	94
	Yes	23	6
Do you have polyps in the nose?	No	355	92
	Yes	31	8
Your family members suffer from polyps in the nose?	No	228	59
	Yes	158	41

Risk factors were compared with the age and gender of the study participants. None of the above-mentioned factors were significantly associated with age, however, having nasal polyps and a family history of polyps showed a significant association with the gender of study participants. The frequency of having nasal polyps was significantly higher among male participants, however, having a family history of nasal polyps was significantly higher among female participants (Table 2).

**Table 2:** Correlation of risk factors with gender and age (*): P-value of less than 0.05

Variable		Gender				Age in years								p-value	
		Female		Male		18-25		26-35		36-45		>45		Gender	Age
		N	%	N	%	N	%	N	%	N	%	N	%		
Distinguish smells	No	10	4	6	5	7	4	5	6	0	0	4	6	0.476	0.238
	Yes	262	96	108	95	168	96	77	94	64	100	61	94		
Nose gets stuffy frequently	No	177	65	67	59	114	65	50	61	41	64	39	60	0.424	0.858
	Yes	95	35	47	41	61	35	32	39	23	36	26	40		
Get cold frequently?	No	217	80	92	81	139	79	63	77	53	83	54	83	0.836	0.740
	Yes	55	20	22	19	36	21	19	23	11	17	11	17		
Do you have asthma?	No	254	93	101	89	159	91	76	93	60	94	60	92	0.114	0.889
	Yes	18	7	13	11	16	9	6	7	4	6	5	8		
Notice any symptoms after taking aspirin?	No	256	94	107	94	167	95	74	90	60	94	62	95	0.922	0.403
	Yes	16	6	7	6	8	5	8	10	4	6	3	5		
Have you ever used cortisone for your nose?	No	236	87	92	81	154	88	64	78	54	84	56	86	0.128	0.219
	Yes	36	13	22	19	21	12	18	22	10	16	9	14		
Have you ever had nose surgery?	No	256	94	101	89	162	93	74	90	61	95	60	92	0.060	0.721
	Yes	16	6	13	11	13	7	8	10	3	5	5	8		
Have you ever had a broken nose?	No	258	95	105	92	165	94	77	94	59	92	62	95	0.298	0.891
	Yes	14	5	9	8	10	6	5	6	5	8	3	5		
Do you have polyps in the nose?	No	256	94	99	87	160	91	75	91	60	94	60	92	0.016*	0.944
	Yes	16	6	15	13	15	9	7	9	4	6	5	8		
Your family members suffer from polyps in the nose?	No	152	56	76	67	115	66	45	55	34	53	34	52	0.049*	0.114
	Yes	120	44	38	33	60	34	37	45	30	47	31	48		

The educational status of study participants showed a significant association with the use of cortisone and having a broken nose, while none of the other factors showed a significant association with the educational status of study participants. Except for one factor, the socioeconomic status (SES) of study participants showed no significant association with other factors. Having a family history of nasal polyps showed a significant association with the socioeconomic status of study participants (Table 3).

**Table 3:** Correlation of risk factors with education and occupation (*): P-value of less than 0.05

	Education				Occupation				P-value	
	Certificate Or less (n)	College (n)	High School (n)	PG (n)	Employed (n)	Retired (n)	Student (n)	Unemployed (n)	Education	SES
Do you have the ability to distinguish smells?										
No	1	10	2	3	5	2	4	5	0.155	0.685
Yes	15	247	87	21	113	28	140	89		
Does the nose get stuffy frequently?										
No	7	161	58	18	77	18	93	56	0.238	0.802
Yes	9	96	31	6	41	12	51	38		
Do you get cold frequently?										
No	10	205	74	20	93	27	115	74	0.284	0.556
Yes	6	52	15	4	25	3	29	20		
Do you have asthma?										
No	15	236	81	23	106	28	132	89	0.880	0.624
Yes	1	21	8	1	12	2	12	5		
Did you notice any symptoms after taking aspirin?										
No	14	242	85	22	107	29	138	89	0.613	0.302
Yes	2	15	4	2	11	1	6	5		
Have you ever used cortisone for your nose?										
No	15	209	82	22	96	26	126	80	0.045*	0.573
Yes	1	48	7	2	22	4	18	14		
Have you ever had nose surgery?										
No	14	236	83	24	107	28	134	88	0.430	0.843
Yes	2	21	6	0	11	2	10	6		
Have you ever had a broken nose?										
No	13	240	88	22	107	29	137	90	0.032*	0.315
Yes	3	17	1	2	11	1	7	4		
Do you have polyps in the nose?										
No	15	237	81	22	109	27	134	85	0.977	0.869
Yes	1	20	8	2	9	3	10	9		
Do any of your family members suffer from polyps in the nose?										
No	11	140	61	16	67	14	99	48	0.081	0.017*
Yes	5	117	28	8	51	16	45	46		

## Discussion

The present study was cross-sectional where the interest was to measure the prevalence of CRS with nasal polyps among Saudis and non-Saudis. Data were collected that included both genders in order, individuals from different age groups, areas (Saudi & non-Saudi), and individuals from different economic backgrounds. This was to ensure the diversity of the sample to capture all possible factors that might contribute to the knowledge of rhinosinusitis with nasal polyps. A substantial link was discovered between chronic rhinosinusitis with nasal polyps and demographic factors. The risk factors were compared with the age and gender of the study participants. A total of 262 female patients and 108 males were able to distinguish smells. Frequently stuffy nose and asthma are positively (r- 0.47) correlated with CRSwNP. Moreover, a broken nose and a family history of nasal polyps both were positively correlated with CRSwNP and significant. However, a study was conducted in 2015 by Stevens et al. on CRSwNP patients having sinus surgery at a tertiary care facility that discovered that females with the condition had a more severe illness than males. CRSwNP was identified in 38% and 62% of males and females, respectively. Females were considerably more likely than males to have radiographic signs of sinus disease, to take systemic corticosteroids at the time of sinus surgery, and to need revision sinus operations [9]. A great number of studies reported about a higher prevalence in the female gender [10,11]. In contrast to that, an investigation performed in Korea identified the male gender as a risk factor for CRS. Possibly the striking differences are also because of the prolonged duration of the disease in men. In terms of prognosis, boys were more likely to have a poorer prognosis regarding the surgical therapy of CRS. The rising health awareness of the female gender associated with a more frequent seeking for medical help could account for a higher disease rate in men caused by a widespread smoking behavior [12]. The high-rise prevalence of younger patients may be a hint to an increasing total incidence of CRS in the future.

The educational status of study participants showed a significant association with the use of cortisone (p-value 0.032). Having a family history of nasal polyps showed a significant (p-value 0.017) association with the socioeconomic status of the study participants.

Nasal polyps are commonly observed in families, pointing to either a genetic component or a common environmental influence. More than half (52%) of 224 individuals with nasal polyps in a study conducted by Rugina et al. had a favorable family history [13], while 14% of family histories in a study done by Greisner and Settipane clearly suggested genetic aspects in the development of nasal polyps [14]. CRS as a result of a ciliary defect such as a primary ciliary dyskinesia plays an important role in the pediatric age group. The primary ciliary dyskinesia can be seen in 5.6% of the children with recurring infections [15]. Apart from the genetic origin of the disease, CRS occurs more often in CRSwNP than in CRSsNP. Cystic fibrosis has an incidence of about 1:2,500 with increasing tendency. In these cases, nasal polyposis was seen in 4-44% of the patients. In the subgroup of those patients who had undergone lung transplantation, CRSwNP was noted in 10%. Inversely, patients with CRSwNP had a gene mutation associated with cystic fibrosis in 6% [16-18]. Another genetic factor for the genesis of CRSwNP is mentioned in a Polish study that found an increased rate of a -765 G/C polymorphism of cyclooxygenase-2 (OF: 4.04; 95% CI: 2.32-7.03) and a C allele (OR: 3.68; 95% CI: 2.38-5.68) compared to the control group of the same gender and age. Further, the study reports about families that allow the conclusion of a still not clear hereditary background of CRSwNP. Possibly HLA-A74 plays a role in this matter. Two different polymorphisms in the field of tumor necrosis factor (TNF)-alpha protein 3 gene are associated with severe CRS. Additionally, three single nucleotide polymorphisms of the IL22RA1 area showed significant differences regarding their existence in patients with severe CRSwNP in comparison to healthy people of the same region [19].

In addition to CRS, bronchial asthma and COPD are related to other diseases that are also linked as risk factors of RS. Those include aspirin-exacerbated respiratory disease, gastro-esophageal reflux and depression. Therefore, the striking association or the common pathophysiology of CRS and bronchial asthma may also account for these and further, newly observed associations. Information on the relation with subtypes of CRS is scarce and mainly concerns CRSwNP: 7%-15% of all asthmatics also complain about CRSwNP. Prospectively, bronchial asthma could be seen in CRSwNP patients more often (23%) than in the control group (6%). In addition, females with CRSwNP develop 1.6 times more often bronchial asthma [20].

Gao et al. revealed the significant associations between occupational and environmental factors and CRS which closely matched with our findings in the categories of employed, retired, and students [21]. There is evidence pointing out that special occupational groups are more likely to develop CRS because of environmental triggers. A Danish subgroup of the GA2LEN study reports a higher prevalence for female blue-collar workers in contrast to white-collar workers (employees and self-employed people). Mainly exposure to dust, gas, smoke, and steam was highlighted as being responsible for their ailment. Information from Lower Saxony confirms a higher percentage of diseases of the lower airways in clustered neighborhoods to large fattening farms [22].

Furthermore, several recent cross-sectional studies exploring the associations, relationships, and correlations with multiple disorders such as diabetes [23], obstructive sleep apnea [24], neurodegenerative dementia [25], and obesity [26] have been identified, but no direct or overlapping associations are correlated. In Canada, the supplementary diagnosis of depression was made more than twice as much (8.4 vs. 4.1%) and additionally, the antidepressive therapy with drugs was conducted (9.1 vs. 4.6%). In addition, psychological treatment is more often prescribed in individuals with CRS (11.8 vs. 7%). Besides, depressive and anxious patients are more sensitive regarding the symptoms of CRS [27]. In a Taiwanese population-based study, not only obesity (adjusted OR: 2.50; 95% CI: 1.90-3.30) but also weight loss (adjusted OR: 2.58; 95% CI: 1.30-5.13) was represented more often in patients with CRS [28].

Palmer et al. conducted a cross-sectional, population-based survey of U.S. adults with symptoms of chronic rhinosinusitis where the interesting fact that women are more likely to suffer than men was found to be quite analogous in our study [29].

The link between antrochoanal polyp (ACP) and allergy yet remains controversial. While the proposed etiology behind ACP is unknown, a connection between ACP and allergic rhinitis or ipsilateral maxillary sinusitis has been noted in the pediatric age group [30].

However, the study has multiple limitations such as no histopathological study included, the reason for gender-oriented disease proliferation is not explored, and understanding and factorial correlation of disease pathophysiology of antrochoanal polyp and ethmoidal nasal polyp is not established.

## Conclusions

In conclusion, nasal polyps are more common in males than females, however, having a family history of nasal polyps was significantly higher among female participants. A comprehensive knowledge of the distinctions between the pathophysiology of the antrochoanal polyp and ethmoidal nasal polyps is still required. There haven't been a lot of actual studies done to identify the variations in their pathophysiology and etiology. In order to fully understand the etiologies behind them and to identify the distinctions, research is being conducted.
